# An Historical Review of Dental Surgery, from the Earliest Period to the Commencement of the Present Century

**Published:** 1859-07

**Authors:** A. Hockley, A. T. L. Jourdain


### SELECTED ARTICLES.

### ARTICLE X.

*An Historical Review of Dental Surgery, from the earliest period to the commencement of the present century.* From Kurt Sprengel's "Geschichte der Medicin" (History of Medicine.) Translated from the French of A. T. L. Jourdain, with critical and explanatory Notes. By A. Hockley.

Some explanation, I consider, is necessary, on presenting to the readers of this Journal a translation, the first into the English language, of that portion of Sprengel's "History of Medicine" which relates to "Dental Surgery."

Thinking the rise and progress of that branch of science to be a subject somewhat interesting, and not entirely unprofitable, I set about collecting the materials necessary for writing its history, from the earliest time to a very recent date; and for that purpose consulted, among others, the above work of Sprengel, which, although a valuable acquisition to medical literature, and frequently referred to by medical and other writers, has never yet been translated into the English language. But finding that the chapter upon operations relating to the teeth contained an historical summary of dental surgery from the earliest period to the commencement of the present century, and, therefore, that my own labors and intentions had been to a great extent anticipated, and, also, that the sources drawn from, were some of those to which I had gone for information, I determined upon a translation of it in preference to completing the task I had commenced, adding some of my own materials in the shape of notes.

I may here observe, that the laudable attempts which are being made to raise the status of the dental profession in this country—the publication of journals devoted to the "diffusion of useful knowledge" bearing directly upon dentistry—and a belief that any addition to the "knowledge" of the history of the science may be made "useful" by those who study it as a profession, have been the only incentives to my appearing in the pages of the *Quarterly Journal of Dental Science.* A. H.

---

As the dental art forms at the present day a distinct branch of surgery, and is cultivated exclusively by one class of practitioners, so also we find, that its practice among the Egyptians, was, from a very remote period, confined to a particular class who devoted themselves entirely to its study.* It was not, however, unknown to the ancient physicians, since it is related that Æsculapius III., the son of Arsippus and Arsinöe, was the first who

* The Egyptians paid great attention to health; and so wisely, says Herodotus, was medicine managed by them, that no doctor was permitted to practice any but his own peculiar branch; some were oculists who only studied diseases of the eye, others attended solely to complaints of the head, others to those of the teeth; some again confined themselves to complaints of the intestines, and others to secret and internal maladies: and it is a singular fact, that their dentists adopted a method not very long practiced in Europe, of stopping teeth with gold, proofs of which have been obtained from some mummies in Thebes. They received certain salaries from the public treasury, and after they had studied those precepts which had been laid down from the experience of their predecessors, they were permitted to practice. Though paid by the government, it was not illegal to receive fees for their advice and attendance.—*Travels in Egypt, by Sir. G. Wilkinson.*

Of the skill of the Egyptians in the manufacture of artificial teeth, there are specimens in the museums of Paris and Berlin. The valuable and extensive collection of Joseph Mayer, Esq., F. R. S., of Liverpool, contains, among other Egyptian antiquities, two pieces of artificial teeth, one of five teeth carved in bone, the other of two teeth in sycamore wood set in gold:—and Dr. Purland has in his collection a tooth found pivoted to a stump in the head of a mummy. Belzoni and others discovered artificial teeth made of sycamore wood, in the sarcophagi of the Egyptians.

practiced the extraction of teeth. In fabulous times, precepts had then been given against the inconsiderate extraction of these small bones; for Erasistratus speaks of a tooth extractor in lead, which was suspended in the Temple of Delphos, to indicate that only teeth which were quite loose should be extracted. Hippocrates also recommends this precept before every other:—"Extract," says he, "those teeth which are decayed and loosened; but when they are neither decayed nor loosened, yet cause violent pains, it is necessary to cauterize them." Heraclides of Tarentum, and Herophilus, relate cases of persons who had died in consequence of the extraction of teeth. Hippocrates mentions, likewise, a remedy against the fetidness of the breath, and discoloration of the teeth; and Menamachus* prescribes one for decayed teeth.

Celsus has given a tolerably complete insight into all the operations relating to the teeth. He advises not to resort too hastily to the extraction of a decayed tooth, unless compelled by imperative circumstances. When violent pains necessitate the removal of the tooth, it may be made to fall in pieces by inserting into its cavity a peppercorn, or ivy berry, or by placing around it a preparation composed of the prickle of the thornback (pastinaca) roasted, and afterwards bruised and mixed with resin. If scissile alum, wrapped in a litle wool, be placed in the cavity, the tooth will be preserved, and the pain removed. The points of a decayed tooth which hurt the tongue must be filed down. Parulides must be completely removed by excision, and ulcers of the gum, when excited by a carious tooth, should be opened, and the tooth extracted. Any spiculæ of bone, of which the tissue may be impaired, must be removed, and the bone afterwards filed down. When the teeth begin to loosen by reason of the weakness of their roots, or the wasting of the gum, the actual cautery should be ap-

*** The preparation which Menamachus composed for the molar teeth, consisted of saffron, cardamom, soot, frankincense, parts of figs, pellitory, and mustard.—*Celsus de Medicina.***

plied to the latter, which should afterwards be anointed with honey; but when extraction is absolutely necessary, the gum must first be detached from the tooth, and the tooth moved repeatedly, until it become thoroughly loosened. The tooth is then, if possible, removed by the fingers, or the forceps, if it cannot be reached otherwise, taking care, nevertheless, if the tooth is decayed, to fill it with a small piece of lint, or lead, so that it may not break from the pressure of the instrument, which must be pulled direct, lest the thin bone, the alveoli, to which the bent fangs adhere, be broken in any part, for the extraction of a firm tooth is attended with the greatest danger, the jaw being sometimes dislocated, or the operation, if performed on the superior maxillary, may communicate a shock to the eyes or temples.

It sometimes happens, especially when the tooth to be extracted is small, that the forceps seizes at the same time a portion of the jaw, and when there is a greater effusion of blood than ordinary, we may discover that some portion of the bone is fractured. In this case, the gum must be incised, and the detached bone removed with small pincers, because, without this precaution, the entire jaw bone would tumefy. After this operation, a poultice, composed of meal and figs, is to be applied, until matter be promoted; the gum is then lanced, and a search made for the splinters. If the bone, without being fractured, is found to be affected by caries, it is filed down.

Celsus scraped a carious tooth until the decayed part was quite removed, and then anointed it with a mixture of rose leaves, nutgalls, and myrrh. He fixed loose teeth by attaching them with a gold thread to those adjoining that were firm, and when in the case of children a second tooth happened to grow before the first had been shed, he extracted the latter, and pressed upon the new tooth so as to force it into its proper position. Such stumps, or portion of roots as remained in the alveolus after the extraction of a tooth, were removed by a forcep, called by the Greeks, Rizagra.

Of the numerous tooth powders invented by the ancients, those of Democritus (of which Galen has given the receipt in verse) and Octavia, sister of Augustus, (found in Scribonius Largus,) are the most distinguished.*

Scribonius, who does little more than publish formulas of everything relating to surgery, here abandons his ordinary empiricism, and gives some very judicious precepts. I know well, says he, that many believe there is no better remedy for toothache than extraction, but before proceeding to that, there are many other remedies that may be successfully employed. It is not necessary to proceed at once to extraction, even though caries exist, for by removing the decayed portion of the tooth with a sharp cutting instrument, such removal causing no pain, whatever remains is as sound as a perfect or healthy tooth.

Violent toothache yields sometimes to various medicaments, as lotions, fumigations, applied to the diseased part. Loose teeth may be tightened by a decoction of patience root in wine or asses milk. Scribonius also mentions various preparations for tooth powders, that are likewise to be found in Galen, a great number of which had been invented by Andromachus, Antiphanus, Aristocrates, Diocles, Criton, and Temocritus.

Archigenes, for the cure of aching teeth, recommends that they be washed with a decoction of gall nuts, or alkekengi berries, in hot vinegar. Into decayed teeth he inserted sulphate of iron and turpentine, or a mixture of

* Galen has transmitted to posterity the formula of two dentifrices, written in verse. Democritus drew them out of a little book called *Pythicus*, which was also the name of him who prepared them; one of them used the powder of radishes dried in the sun, or of white glass finely pulverized and mixed with Indian spikenard. Messalinus made use of a dentifrice composed of burnt hartshorn, mastic, and sal ammoniac. Perhaps it is doubtful whether these compositions were preferable to the dentifrice which was presented to Calpurnianus by Apulias: the charming verses by which it was accompanied, announced it to be composed of the finest drugs of Arabia. "It is an excellent powder," says he, "very fine, which has the property of whitening the teeth, of dissipating the swelling of the gums, and of removing that which adheres to them so completely, that no traces of tartar can be perceived, even when the gums are exposed by laughter."—*Advice of the ancient Poets, by J. R. Duval.*

pepper and oil of almonds, of which he also poured a few drops into the ear. Teeth slightly inflamed and changed in color, he anointed with a mixture of carbonate of soda, peach stones, and resin, until the pain had ceased. He mentions several methods to prevent bleeding of the gums. Appolonius Migmatopolus has made known similar preparations, and marked out several precepts relative to the employment of vapor baths, and cauterization of decayed teeth. Injections of the nose and ears he considers proper to cure toothache.

Galen demonstrates that the teeth are not entirely deprived of sensibility, as might be inferred from the absence of pain when they are filed, from the following facts; having proved on himself that the pain proceeds from the small nerves enclosed by the roots—that the substance of the teeth may easily become inflamed, as denoted by the throbbing pain that is felt, and the blackish color they acquire, and the efficacy of antiphlogistics in removing these complaints. As the vacillation of the teeth, observed among aged persons, does not depend on the teeth themselves, but upon the parts surrounding them, being caused, in fact, by their imperfect nourishment, Galen indicates no other remedy than the strengthening the gums. He likewise, with much sagacity, advises, that, before we attempt to cure toothache, we should endeavor, above all, to become acquainted with the cause of it, as if we can succeed in removing that, the pains appease themselves. Dental caries, in a great number of cases, proceeds equally from internal causes. Galen extols above everything, as a remedy for toothache, the use of vapor baths, and the introduction into the tooth of a small piece of wax, pressed well in by means of a probe. When the pain cannot be appeased, the tooth should be perforated with a small trephine, and the same remedies applied as before: if ultimately this method fails, and it is considered desirable to remove the tooth, it can be done without pain to the patient by the application of pyrethrin root and strong vinegar, from the action of which, the remaining teeth may be preserved by covering them with a layer of wax. At the expiration of an hour, the tooth becomes so loosened, as to be easily removed. It will also fall out by itself if placed in contact with sulphate of copper and strong vinegar.

Black hellebore, or ginger, inserted in the tooth, removes the pain, and prevents fetidity of the breath. When from a blow or otherwise a tooth is loosened, and projects beyond the level of the others, the projecting part should be filed down, for which purpose a small file is used, and the tooth is held between the fingers, so as to prevent its being further loosened: as soon as the operation causes pain, it is suspended, antidinics are administered, and after the lapse of a few days it is repeated.

Finally, Galen, whilst agreeing with his contemporaries that it was possible to cause a tooth to fall in pieces, and that without pain; at the same time advises in one of his writings, that in extracting teeth, it is necessary always to commence the operation by cutting around the gum.

According to Cœlius Aurelianus, scarifications of the gums, and the application of cupping glasses to the cheek, are frequently a great relief in toothache. This complaint may also be combatted by a sort of cauterization which is applied by twisting some wool around a probe, dipping it into boiling oil, and applying it either to the tooth itself, or on both sides of the gum.

Cœlius condemns the inconsiderate extraction of teeth, which, as it removes the pain without curing it, ought only to be regarded as a last resource when every other has failed; he urges, above all things, that care should be taken in extracting teeth which are firmly fixed, as the adjacent parts, particularly the eyes, are always affected by the operation, and frequently it is not only one tooth, but the entire jaw which aches in such a manner as to render it necessary to extract all the teeth in order to obtain relief.

Marcellus of Bordeaux was considerably behind his predecessors, whose precepts, as we have seen, were generally very judicious. This writer repeats the same words of Scribonius, and recommends the use of amulets and other superstitious means. Thus, when a tooth which is loose or painful is to be extracted, the nose of the patient should be rubbed with brown sugar, ivy, and green oil: he is advised to hold his breath, a stone is then placed between his teeth, and he is made to close his mouth. The fluid which causes the pain is then seen to flow from the mouth in such quantity as frequently to fill three pots; after having cleansed the nose with pure oil, and rinsed the mouth with wine, the tooth is no longer painful, and may be easily extracted. The tooth will likewise drop out by itself if the tooth be rubbed with African sponge, but care must be taken not to meddle with a tooth which is healthy. To preserve oneself to a certainty from the toothache, it is only necessary, at the return of the swallow, to repair silently to the border of a clear rivulet, to take some water in the mouth, and to rub the teeth with the forefinger of both hands, and repeat these words:—"Hirudo, tibi dico, quo modo hoc in nostro iterum non erit, sic mihi dentes non doleant toto anno."

The teeth, says Ætius, are open at the roots, and these openings admit small nerves coming from the tri-facial. For this reason they are the only bones which can become painful of themselves; they are nourished by the deposition of a superfluous nervous fluid in their interior; but at the period of old age the process of nutrition ceases, they then become loose and finally drop out. Thus, there are two kinds of odontalgia, the superabundance, or want of the nutritive principle, and by either of these we should be guided in the choice of remedies. These remarks of Ætius display a skillful anatomist for the time in which he lived, as those of Galen, on the inflammation of the teeth, denote a physician well versed in diagnosis. Ætius advocates the burning of phagedenic ulcers of the teeth, with a probe covered with wool and dipped into boiling oil ; also the excision of abscesses of the gums, because when an incision only is made, ulcers frequently result from it. When the epulis does not yield promptly to caustics, it is seized with pincers and excised with a straight bistoury. When incision does not suffice to cure ulcers of the gum, it is best to extract the decayed tooth which occasions them. As to the manner of filing and extracting the teeth themselves, as well as the treatment of caries with which they may be affected, Ætius does nothing but copy Galen.*

* With the exception of Galen and Ætius, the knowledge of the Greek and Roman writers of the structure and development of the teeth was very limited, and their views upon the subject extremely fanciful and imperfect. Hippocrates describes the teeth as a glutinous substance, growing from the head and jaws, the fatty part of which is dried by heat, aud burnt up, and that they are much harder than the other bones, because there is nothing cold in them. That in the fœtus they are nourished by the food of the mother, and after birth, by the milk which the infant sucks from the breast. He considers that to have many teeth is a sign of living to a considerable age. Aristotle asserts that the teeth continue to grow and increase in length during life, which constitutes the difference between those and the other bones of the body. That man has more teeth than woman, and that the same difference may be observed between the sexes of various classes of animals. Aretæus considers that the cause of toothache is known only to God; and Pliny remarks on the indestructibility of these organs, that while every other part of the bodies enclosed in the sarcophagi was destroyed, the teeth remained perfect. Galen, who possessed a greater amount of knowledge on the subject than any other ancient writer, enters into a full description of the structure and formation of the teeth. He defines them as true bones, and states that they are formed during the period of gestation, but remain hidden in the alveoli until birth. That the canine teeth had received the appellation of ocular, because branches are sent to them from a nerve, which distributes others to the eye; and he describes with accuracy the forms of the roots of the molar teeth of both jaws.

It appears strange that the Greek and Roman writers make no mention of the replacement of the natural teeth by artificial ones, as at the period they wrote, and for a considerable time previously, this branch of the dental art was extensively practiced at Rome and Athens, as may be inferred from the allusions of Martial and Catullus, and other satirical writers to the manners and customs of the people.

Celsus, who describes most accurately the various operations in dental surgery, speaks only of the fixing of teeth, which are loosened, to the adjoining ones by means of gold or silver wire; a method adopted nearly 500 years previously by Hippocrates, and recommended by him chiefly for adjusting fractures of the lower jaw. And who advises, that when the broken ends of the bone are placed in apposition, the teeth should, if they be disturbed and loosened, be tied to others with a gold thread. This practice of fixing teeth was not an uncommon one at that period, as the following extract from the celebrated Laws of the

Paulus Ægineta distinguishes very well epulis, a fleshy excrescence of the gum, growing above the teeth, from parulis, an abscess of the same gum. The former is removed by raising it with a hook or forceps, and cutting it out with a bistoury, and the latter is cut round with a sharp-cutting instrument, although it may sometimes be cured by making a simple incision. After the operation, the patient's mouth should be rinsed with wine, and a mixture of honey and vinegar, followed by an application of verdigris. When a tooth is to be extracted, the gum must be detached from it, down to the edge of the alveolus. It is then seized with the Rizagra, and well shaken, in order to loosen it, and afterwards extracted by pulling it perpendicularly. He also, with Celsus, recommends that previous to the operation, the cavity of the tooth be filled with a small tent, so that the tooth may not be broken; projecting teeth may be filed, and the same means may be adopted for removing the sharp edges of those that have been broken; Paul admits with Galen the inflammation of the teeth, and recommends various electuaries and powders, for this, as well as for several other diseases.—*Lond. Dent. Review.*

(To be continued.)

Twelve Tables, will show: the tenth of which, relating exclusively to burials and funeral ceremonies, among other directions, says—"Let no gold be used, but if any one has had his teeth fastened with gold, let it be lawful to bury or burn that gold with the body."

"The advice of the ancient poets upon the preservation of the teeth," formed the subject of a paper read in 1803, by J. R. Duval, before the Society of Medicine, of Paris. An English translation of the same, by J. Atkinson, under the title of "The Dentiste de la Jeunesse," was published in London, 1820: in it will be found many allusions to the wearing of artificial teeth by the ancients.

